# Clinical and molecular characterization of a large primary hyperoxaluria cohort from Saudi Arabia: a retrospective study

**DOI:** 10.1007/s00467-022-05784-y

**Published:** 2022-11-21

**Authors:** Majid Alfadhel, Muhammad Umair, Malak A. Alghamdi, Khalid Al Fakeeh, Abdullah T. Al Qahtani, Afrah Farahat, Mohamed A. Shalaby, Jameela A. Kari, Rupesh Raina, Pierre Cochat, Khalid A. Alhasan

**Affiliations:** 1 Genetics and Precision Medicine Department (GPM), King Abdullah Specialized Children’s Hospital, King Abdulaziz Medical City, Ministry of National Guard Health Affairs (MNG-HA), Riyadh, Saudi Arabia; 2 Medical Genomic Research Department, King Abdullah International Medical Research Center(KAIMRC), King Saud Bin Abdulaziz University for Health Sciences(KSAU-HS), Ministry of National Guard Health Affairs (MNG-HA), Riyadh, Saudi Arabia; 3 Medical Genetic Division, Department of Pediatrics, College of Medicine, King Saud University, Riyadh, Saudi Arabia; 4 Nephrology Division, Department of Pediatrics, King Abdullah International Medical Research Center (KAIMRC), King Saud Bin Abdulaziz University for Health Sciences (KSAU-HS), Ministry of National Guard Health Affairs (MNG-HA), Riyadh, Saudi Arabia; 5 Division of Nephrology, Department of Pediatrics, King Saud University Medical City, King Saud University, Riyadh, Saudi Arabia; 6 Pediatric Nephrology Center of Excellence, King Abdulaziz University Hospital, Department of Pediatrics, King Abdulaziz University, Jeddah, Saudi Arabia; 7 Department of Nephrology, Cleveland Clinic Akron General and Akron Childrens Hospital, Akron, OH USA; 8 Centre de Référence Des Maladies Rénales Rares Néphrogones, Hospices Civils de Lyon & Université Claude-Bernard Lyon 1, Lyon, France; 9 Division of Nephrology, Department of Pediatrics, College of Medicine, King Saud University, Riyadh, Saudi Arabia; 10 Division of Pediatric Kidney Transplant, Organ Transplant Center of Excellence, King Faisal Specialist Hospital and Research Center, Riyadh, Saudi Arabia

**Keywords:** Saudi Arabia, Primary hyperoxaluria, *AGXT*, *GRHPR*, Nephrocalcinosis

## Abstract

**Background:**

Primary hyperoxalurias (PHs) constitute rare disorders resulting in abnormal glyoxalate metabolism. PH-associated phenotypes range from progressive nephrocalcinosis and/or recurrent urolithiasis to early kidney failure.

**Methods:**

A retrospective study was conducted for patients with confirmed PH diagnoses from three tertiary centers in Saudi Arabia. Detailed clinical molecular diagnosis was performed for 25 affected individuals. Whole exome sequencing (WES)–based molecular diagnosis was performed for all affected individuals.

**Results:**

The male:female ratio was 52% male (*n* = 13) and 48% female (*n* = 12), and consanguinity was present in 88%. Nephrolithiasis and/or nephrocalcinosis were present in all patients. Kidney stones were present in 72%, nephrocalcinosis in 60%, hematuria in 32%, proteinuria in 16%, abdominal pain in 36%, developmental delay in 8%, and chronic kidney disease stage 5 (CKD stage 5) was observed in 28% of the patients. The most common PH disorder was type I caused by variants in the *AGXT* gene, accounting for 56%. The *GRHPR* gene variants were identified in 4 patients, 16% of the total cases. Seven patients did not reveal any associated variants. Missense variants were the most commonly observed variants (48%), followed by frame-shift duplication variants (28%).

**Conclusions:**

Characterization of the genetic and clinical aspects of PH in this unique population provides direction for improved patient management and further research.

**Graphical abstract:**

**Figure:**
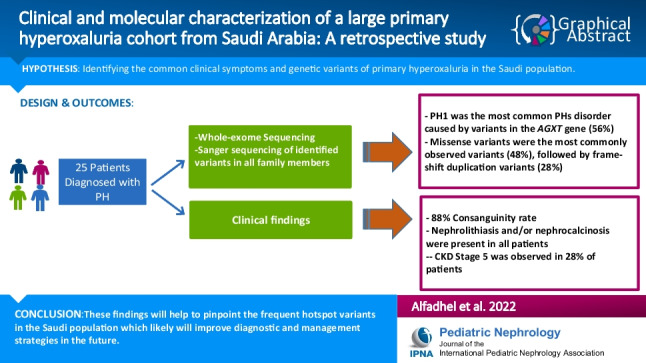
A higher resolution version of the Graphical abstract is available as Supplementary information

**Supplementary Information:**

The online version contains supplementary material available at 10.1007/s00467-022-05784-y.

## Introduction

Primary hyperoxalurias (PHs) are rare inborn errors of metabolism (glyoxylate) which lead to the overproduction of oxalate in the liver [1, 2]. The accumulation of oxalate results in the calcium oxalate (CaOx) crystals that are deposited in the urinary tract and kidneys [3]. Hyperoxalurias are classified into two main categories (PHs and SHs) [4, 5]. Secondary hyperoxalurias (SHs) generate from an increased oxalate absorption due to various alterations, including intestinal inflammatory diseases, very low calcium diet, bariatric surgery, or undue oxalate precursors intake [6, 7].

PH type I (OMIM 604,285) is the most severe type of hyperoxaluria. It is an autosomal recessive disease resulting in an insufficiency of alanine glyoxylate aminotransferase enzyme (AGT), which takes part in glyoxylate metabolism final step [8]. As a result, glyoxylate is not effectively metabolized to glycine, causing overproduction of both glycolate and oxalate [9]. PH type I is associated with progressive nephrocalcinosis, or recurrent urolithiasis, which leads to CKD stage 5 during the first 2–3 decades of life [10]. Infantile oxalosis is the most severe clinical presentation of PH type I.

PH type II (OMIM 260,000) results from the deficiency of the enzyme glyoxylate reductase/hydroxypyruvate reductase (GRHPR), which plays a key role in the gluconeogenic pathway and helps in the removal of cytosolic glyoxylate [6, 11]. Lack of GRHPR results in glyoxylate and hydroxypyruvate accumulation that is processed by lactate dehydrogenase to L-glycerate and oxalate. The clinical picture of PH type II is milder compared to PH type I; however, affected individuals may suffer from frequent episodes of urolithiasis that might lead to kidney failure [12].

PH III (OMIM 613,616) results from recessive variants in the *HOGA1* gene, which encodes the enzyme 4-hydroxy-2-oxoglutarate aldolase (HOGA). Clinical presentations include a less severe form with early onset, a low chronic kidney disease risk, an early disease onset, impaired kidney function and recurrent stone disease [13].

In this study, we sought to characterize the genetic and clinical characteristics of PH patients in Saudi Arabia in order to provide guidance to clinicians and researchers interested in this rare disorder.

## Materials and methods

### Inclusion criteria

The study included cases with confirmed molecular diagnoses having PHs type I, II, or III. In addition, cases that presented pathogenic or likely pathogenic variants in either *AGXT*, *GRHPR*, or *HOGA1* genes according to the ACMG criteria were included. Finally, affected individuals having a variant of uncertain significance (VUS) were also included, who had PHs before molecular testing and who had consistent PH phenotypes.

### Genetic testing

The molecular diagnosis in PHs patients was performed using single gene testing or WES, as previously reported [14–16].

### Variant classifications and prioritization

ACMG guidelines were followed for variant classification [17]. Variant filtration was performed using standard methods [18]. Different online databases (ExAC, gnomAD, 1000 genomes, etc.) were used to exclude the polymorphic nature of the identified variants. In addition, the variants were fully segregated within available family members.

### Molecular biogenetic testing

Sanger sequencing was performed to segregate the identified variant in all members of the families, followed by WES. Primers were designed using Primer3 and Sanger sequencing was performed using standard methods.

### Pathogenicity of the variants

Sanger sequencing confirmed the segregation of all the discovered variants. For missense variants, in silico analysis tools, including Polyphen2, SIFT, Varsome and MutationTaster were used to predict the pathogenicity of the novel variants. Conservation of the variants was investigated using HomoloGene (https://www.ncbi.nlm.nih.gov/homologene).

## Results

This retrospective chart review study includes 25 patients diagnosed with PHs from three tertiary centers in Riyadh and Jeddah, Saudi Arabia (October 2019–October 2021). The IRB of KAIMRC, KSU-Medical City Hospital, (Riyadh), and KAU (Jeddah) KSA approved the study. Since the study is based on retrospective data, written informed consent was not required.

### Demographic details of patients

The study consisted of 25 patients with 13 males and 12 females, and affected individuals ranged from 3 to 50 years of age (Table 1). The majority (96%) of participants (24/25) were less than 18 years old. All of the affected individuals were from different regions of Saudi Arabia [North (7/25: 28%), central (13/25: 52%), West (5/25: 20%)]. Consanguinity was reported in 88% of the cases (22/25). The age at diagnosis of the affected individuals was between 6 months and 39 years, and a positive family history was observed in 32% (8/25) of cases.

**Table 1:** Clinical description of 25 PH patients investigated in the present study

Patient #	Gender	Positive family history	Consanguinity	Bilateral kidney stone	CKD Stage 5	Nephrocalcinosis	Failure to thrive	Skeletal manifestations	Hematuria	Proteinuria	Dysuria	Abdominal pain	Developmental delay (Gross/Fine motor)	Language problems	Abnormal ultrasound	Skeletal malformation	Dialysis	Liver transplant	Deceased
1	F	+	+	+	+	+	−	+	−	−	−	−	−	−	+	−	** + **	+	-
2	M	+	+	+	+	+	−	+	−	−	−	+	−	−	+	+	** + **	−	-
3	M	+	+	+	−	+	−	−	+	−	−	−	−	−	+	−	−	+	-
4	F	+	+	+	+	+	−	+	−	−	−	−	+	+	+	+	** + **	−	+
5	F	+	+	−	−	−	−	−	−	+	−	+	−	−	+	−	−	−	-
6	M	−	+	−	−	−	−	−	−	−	−	−	−	−	+	−	−	−	-
7	M	−	−	−	−	−	−	−	+	−	−	−	−	−	+	−	−	−	-
8	M	−	+	+	+	−	−	−	−	−	−	+	−	−	+	−	+	−	-
9	M	−	+	+	−	+	−	−	+	+	−	+	−	−	+	−	−	−	-
10	M	−	+	+	−	+	−	−	−	−	−	−	−	−	+	−	−	−	-
11	F	−	+	+	−	+	−	−	−	−	−	−	−	−	+	−	−	−	-
12	F	−	−	−	−	+	−	−	−	−	−	−	−	−	+	−	−	−	-
13	F	−	+	−	−	+	−	−	−	−	−	−	−	−	+	−	−	−	+
14	M	−	+	+	−	+	−	−	−	−	−	−	−	−	+	−	−	−	-
15	M	−	+	+	−	+	−	−	−	−	−	−	−	−	+	−	−	−	-
16	F	−	−	+	−	+	−	−	−	−	−	−	−	−	+	−	−	−	-
17	F	−	+	−	−	−	−	−	−	−	−	+	−	−	+	−	−	−	-
18	F	+	+	+	−	−	−	−	+	−	−	+	−	−	+	−	−	−	-
19	M	+	+	+	−	−	−	−	+	−	−	−	−	−	+	−	−	−	-
20	M	−	+	−	−	−	−	−	−	−	−	−	−	−	−	−	−	−	-
21	M	−	+	+	−	−	−	−	−	−	+	+	−	−	+	−	−	−	-
22	F	−	+	−	+	+	+	+	+	+	−	−	+	+	−	−	** + **	−	+
23	M	+	+	+	+	+	+	+	+	+	+	+	−	−	+	−	** + **	−	-
24	F	−	+	+	+	−	−	+	+	−	−	+	−	−	+	−	+	−	-
25	F	−	+	+	−	+	−	−	−	−	−	−	−	−	−	−	−	−	-

### Initial symptoms

In most cases, the affected individuals presented with vomiting, flank pain, abdominal pain, kidney stones, hematuria, fever, and nephrocalcinosis.

### Developmental milestones

Of the 25 patients evaluated, 2 (8%) manifested failure to thrive and developmental delay, including delayed gross and fine motor development, and language issues. All of the other affected individuals achieved normal developmental milestones. Three of the affected individuals were deceased (3/25; 12%) at the ages of 13 years, 39 months and 11 months. Cause of death included burden of CKD stage 5 for the youngest patient — the patient’s parents chose to give the child palliative care at home. Thus, the patient died at home with no documented hospital death report. The second patient died due to septic shock, and the third died at the age of 11 months having primary hyperoxaluria and Down syndrome.

### Kidney examination

Detailed clinical investigation showed kidney manifestations in all of the affected individuals, including nephrocalcinosis, hematuria, and multiple kidney stones (Table 1). Nephrolithiasis and/or nephrocalcinosis were present in all patients. Kidney stones were present in (18/25; 72%), while nephrocalcinosis was present in (15/25; 60%), hematuria (8/25; 32%), proteinuria (4/25; 16%), and abdominal pain (9/25; 36%). The age of the patients when kidney stones were observed ranged between 22 months and 13 years, and most of the patients suffered from bilateral kidney stones (18/25; 72%). CKD stage 5 was observed in 28% of the patients (7/25). Skeletal manifestations affected 24% (6/25) of the patients, while other manifestations included recurrent urinary tract infection (12%; 3/25) (Tables 1 and 2).

**Table 2:** Summary of additional clinical features observed in the present cohort

Patient #	Current age	Age at first symptoms	Age at diagnosis	Delay in diagnosis	Symptoms at diagnosis	Symptoms at time of FUP	Variant identified	Treatment	Dialysis	eGFR (ml/min/1.73 m^2^) at diagnosis**
1	13y	**7y, 6mo**	**7y, 8mo**	**2mo**	Vomiting, Bilateral kidney Stone	Elevated Creatinine, Echogenic small kidneys, Buffiness and Hypertension	*AGXT*: c.33dup; p.Lys12GlnfsX156	Pyridoxine, tacrolimus	HD,5–6 sessions per week	**10**
2	15y	**5y**	**11y**	**6y**	Left Flank Pain, Nausea and Vomiting, Bilateral kidney Stone	Recurrent UTI and Multiple Stones	*AGXT*: c.33dup; p.Lys12GlnfsX156	Amlodipine, atenolol, Cholecalciferol, Colchicine	HD,5–6 sessions per week	**54**
3	11y	Asymptomatic*	**6y, 1mo**	**N.A**	Bilateral kidney Stone	N.A	*AGXT*: c.187G > C; p.Gly63Arg	Folic Acid	N.D	**91**
4	13y dead	**3y**	**3y, 3mo**	**3mo**	Neprocalcinosis, Bilateral kidney Stone	Neprocalcinosis	*AGXT*: c.187G > C; p.Gly63Arg	Pyridoxine	HD,5–6 sessions per week	**09**
5	8y	**7y**	**8y, 6mo**	**1y, 6mo**	Flank Pain	N.A	*AGXT*: c.33dup; p.Lys12GlnfsX156	N.A	N.D	**120**
6	11y	**5y**	**5y, 1mo**	**1mo**	Flank Pain, Unilateral kidney Stone	Kidney Stone	Not detected	N.A	N.D	**120**
7	3y	**1.5 mo**	**1y. 2mo**	**1y, 0.5 m**	Hematuria	N.A	Not detected	N.A	N.D	**120**
8	13y	**4y**	**9y, 8mo**	**5y, 8mo**	Flank Pain, Bilateral kidney Stone	Hematuria	*GRHPR*: c.707A > T; p.Glu236Val	Pyridoxine, hydrochlorothiazide	HD,5–6 sessions per week	**130**
9	12y	**2y**	**10y**	**8y**	Loin pain, Vomiting	Hematuria, Protenuria	Not detected	N.A	N.D	**120**
10	11y	**2y**	**2y, 4mo**	**4mo**	Acute liver failure with multiple bilateral kidney stones	Acute liver failure	*AGXT*: c.187G > C; p.Gly63Arg	Acetaminophen, Prednisolone	N.D	**87**
11	16y	**04mo**	**3y.2mo**	**2y,8mo**	Bilateral kidney stones, pain	N.A	*AGXT*: c.187G > C; p.Gly63Arg	Pyrodixine, polystra	N.D	**55**
12	9y	**1y**	**2y, 2mo**	**1y, 2mo**	Vomiting, fever, abdominal pain, unilateral kidney stones	Grade 2–3 left side hydronephrosis with small non obstructive kidney stone	*AGXT*: c.187G > C; p.Gly63Arg	Pyridoxine, polycitra, rantidine	N.D	**102**
13	11mo dead	**05mo**	**06mo**	**1mo**	Cognestive heart disease, AVSD, unilateral kidney stones	Stable bilateral medullary nephrocalcinosis. Grade 1 left-sided hydronephrosis	*AGXT*: c.187G > C; p.Gly63Arg	N.A	N.D	**5.4**
14	21y	**6y**	**14y**	**8y**	Bilateral kidney stones	Neprocalcinosis	*AGXT*: c.33dup; p.Lys12GlnfsX156	Pyrodixine and polystra	ND	**99**
15	7y	**01mo**	**1y, 2mo**	**1y, 1mo**	Medullary nephrocalcinosis, Bilateral kidney stones	N.A	*AGXT*: c.33dup; p.Lys12GlnfsX156	Pyrodixine and polystra	N.D	**86**
16	14y	**4y, 8mo**	**5y, 6mo**	**11mo**	Bilateral non obstructive kidney stones	Both kidney have normal corticomedullary differentiation without hydronephrosis of focal lesion	*AGXT*: c.121G > A; p.Gly41Arg, c.865C > T; p.Arg289Cy	Pyrodixine and polystra	N.D	**107**
17	9y. 8mo	**8y**	**9y**	**1y**	Unilateral kidney stones	Kidney colic and UTI	*GRHPR*: c.707A > T; p.Glu236Val	Pyrodixine and polystra	N.D	**130**
18	20y	**14y**	**19y**	**5y**	Left flank pain, Bilateral kidney stones	N.A	*GRHPR*: c.707A > T; p.Glu236Val	Pyrodixine and polystra	N.D	**110**
19	4y	**1y, 10mo**	**2y, 9mo**	**11mo**	Fresh blood in the diaper, Bilateral kidney stones	Blood in urine	Not detected	Pyrodixine and polystra	N.D	**150**
20	8y	**12y**	**18y**	**6y**	Kidney stones	N.A	Not detected	N.A	N.D	**130**
21	50y	**20y**	**39y**	**19y**	Right kidney colic pain, Bilateral kidney stones	N.A	Not detected	Pyridoxine	N.D	**130**
22	3y. 3mo dead	**04mo**	**1y, 4mo**	**1y**	Severe dehydration, kidney failure	Severe dehydration	*AGXT*: c.33dup; p.Lys12GlnfsX156	Pyrodixine and polystra	Initially PD then HD,5–6 sessions per week	**68**
23	11	**3mo**	**3y**	**2y, 9mo**	Hematuria, Bilateral kidney stones	Hematuria	*AGXT*: c.33dup; p.Lys12GlnfsX156	N.A	HD,5–6 sessions per week	**10**
24	9	**3y**	**8y**	**5y**	Kidney pain, Bilateral kidney stones	Hematuria	*HOGA1*: c.860G > T; p.Gly287Val	N.A	HD,5–6 sessions per week	**08**
25	18	**4y**	**8y**	**4y**	Flank Pain, Bilateral kidney stones	N.A	*GRHPR*: c.707A > T; p.Glu236Val	Potassium citrate	N.D	**95**

*HD*, hemodialysis; *PD*, peritoneal dialysis; *N.A*, not available; *N.D.*, not done; *UTI*, urinary tract infection; *AVSD*, atrioventricular septal defect

^*^Patient 3 is asymptomatic and brother of patient 4

**Chronic Kidney diseases (CKD) stages as follow: stage 1: eGFR ≥90; normal or high; stage 2: eGFR: 60-89: mildly decreased; stage 3: eGFR: 30-59: moderately decreased; stage 4: eGFR: 15-29: severely decreased; stage 5: eGFR: <15: kidney failure

### Laboratory examination

Detailed laboratory investigations were performed as summarized in Table 1. Urinary oxalate concentration was investigated in 6 affected individuals (upper normal limit 0.5 mmol/L): all had elevated urinary oxalate level. Plasma oxalate concentration (normal range: 1.3–3.1 μmol/L) was measured in 10 affected individuals and all had high plasma oxalate levels. Three of the affected individuals showed both proteinuria and hematuria, and three affected individuals showed hypercalciuria (Table 1).

### Other abnormalities

Two patients revealed additional clinical phenotypes such as hepatomegaly, skeletal malformation and hypotonia (2/25; 8%).

### Molecular analysis

All of the 25 affected individuals were subjected to single gene testing or whole-exome sequencing (WES) using standard methods, followed by segregation analysis using Sanger sequencing [15]. Molecular genetic testing revealed a confirmed molecular diagnosis in 19/25 cases (76%), and the majority of them were previously reported disease-causing variants. Nearly all identified variants were homozygous and thus demonstrated an autosomal recessive pattern. In addition, one proband revealed an *AGXT*-compound heterozygous variant.

**Fig. 1:**
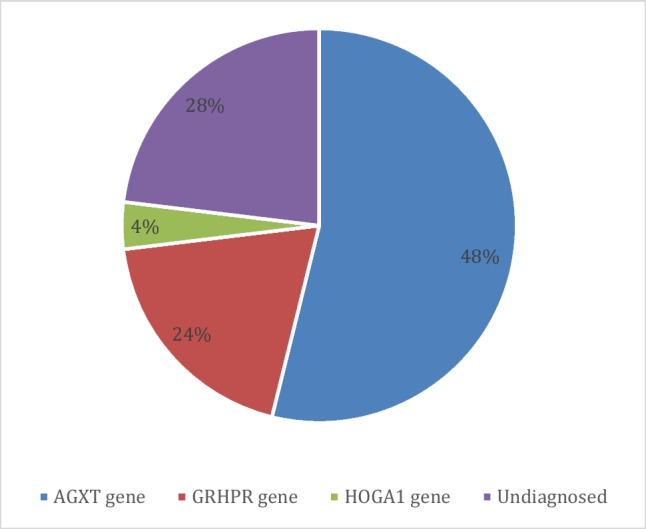
Variants identified in the present study

*AGXT* gene variants [c.33dup; p.(Lys12GlnfsX156), c.187G > C; p.(Gly63Arg)] were observed in 14 cases (56%), *GRHPR* gene variants [c.707A > T; p.(Glu236Val)] were observed in 4 patients (16%), an already reported *HOGA1* gene variant (c.860G > T; p.Gly287Val) was observed in 1 patient (4%), and six patients had negative molecular genetics results (24%). The homozygous variant identified in the *GRHPR* gene (c.707A > T; p.(Glu236Val) was novel. The most frequently identified variants were missense (12/25; 48%), followed by frame-shift duplication variant (7/25; 28%) (Fig. 1). Due to clinical heterogeneity, no clear genotype–phenotype correlations could be concluded. All the identified variants in patients were also screened in all the available family members using standard Sanger sequencing.

The patients with kidney stones and negative molecular results were diagnosed based on the occurrence of kidney stones, type of kidney stones (Ca-oxalate in stone analysis), elevated urine glycolic and oxalic acids in urine organic acid, and elevated urine oxalate /creatinine ratio (hyperoxalemia).

### Management

Dialysis was performed for seven patients (7/25; 28%). Six patients were on hemodialysis (HD), and one was initially on peritoneal dialysis then shifted to HD once referred to tertiary hospital. Depending on feasibility, HD was performed almost 5–6 sessions per week for all patients. Every patient was prescribed different treatments depending on the disease severity and condition, including pyridoxine, polystra tacrolimus, amlodipine, atenolol, cholecalciferol, colchicine, folic acid, hydrochlorothiazide, acetaminophen, prednisolone, and potassium citrate (Table 2). These medications improved the overall quality of life of the affected individuals. In addition, liver transplant was performed for two affected individuals (2/25; 8%) and the eGFR values at the time of liver transplant for patient 1 and patient 3 were 7 ml/min/1.73 m^2^ and 91 ml/min/1.73 m^2^, respectively.

Vitamin B6 (pyridoxine or pyridoxal phosphate) can help to reduce the production of oxalate in some patients affected with PH1 by enhancing the activity of the pyridoxal phosphate-dependent enzyme alanine: glyoxylate aminotransferase (AGT) [19]. Around 30–50% of PH1-affected individuals are pyridoxine-responsive, and after a minimum of 3 months, therapy resulted in a 30% reduction in urine oxalate concentration [20]. For deficient AGT to respond to pyridoxine, some degree of enzyme activity must be preserved that occurs with certain pathogenic *AGXT* variants [21]. Most pyridoxine-responsive individuals show maximum benefit at a dose of 5–8 mg/kg/day [22, 23]. Some require higher doses depending on the disease severity; however, a starting pyridoxine dose of 5 mg/kg/day is recommended. The conditions required for pyridoxine follow-up urine collection include stepwise increase in pyridoxine dose (maximum 10–20 mg/kg/day) [24, 25]. Vitamin B6 response can be difficult to determine in patients with CKD at advanced stage since, at that time, the urinary oxalate excretion rates may be influenced by the low GFR.

## Discussion

Herein, we present the results of a retrospective study of a pediatric cohort of patients with PH from Saudi Arabia. Genetic variants were identified in 76% of patients (19/25). All patients with PH become symptomatic within the first decade of life. The majority of the PH type I affected individuals developed symptoms by 5 years of age; 1 to 2% of infants with PH type I developed kidney failure [26]. On the other hand, 3.2 years to 8.0 years was the average age for the onset of PH type II [12, 27].

While in European studies, PH type II is estimated to range from 1 to 3 in 1,000,000 people [19], the prevalence of PHs in Saudi Arabia is unknown. PH type I is estimated to have a 1:149,000 prevalence in Europeans and 1:157,000 in African Americans [28]. There is no estimated prevalence for PH type III; however, its prevalence is lower than PH type I and type II, making genotype–phenotype correlations difficult.

PHs type I–III are recessively inherited glyoxylate metabolism disorders, associated with an increased endogenetic oxalate synthesis and thus cause excessive accumulation of glyoxylate in the body. Most cases (70–80%) are classified as PH type I [24], caused by disease-causing variants in the *AGXT* gene that results in the absence or deficiency of the AGT enzyme. As transamination of glyoxylate to glycine is catalyzed by AGT, its deficiency allows reduction of glyoxylate into glycolate and this is then oxidized into oxalate. PH type II is less severe, resulting from the deficiency of GRHPR enzyme [1, 12, 24]. Garrelfs et al. [29] reported 101 patients from eleven countries exhibiting PH type II. A total of 82.8% of the patients in their cohort revealed urolithiasis and molecular diagnosis revealed 18 novel *GRHPR* gene mutations. Eight patients had CKD stage 2 and 22 showed CKD stage 5. Fifteen kidney transplantations were also performed in 11 patients [12]. PH type III is caused by disease-causing variants in *HOGA1*, encoding HOGA responsible for mobilizing the last step in hydroxyproline metabolism [19, 24–30].

The disease‐causing variants identified in our study are located in three different genes that have been previously implicated in causing PHs. According to the molecular study results, PH type I (OMIM 259,900) caused by homozygous variants in the *AGXT* gene (OMIM 604,285) was the most common gene mutated. The most common variant and probably the hotspot variant in this region is the frameshift variant in *AGXT* [c.33dup; p.(Lys12Glnfs*156)].

The present study reports 19 variants in 25 different individuals. The rate of previously reported variants associated with PH was high, while the *GRHPR* novel variant (c.707A > T; p.(Glu236Val) causing PH type II was identified in 4 different affected individuals. All of the identified variants in patients were also screened in all the available family members using standard Sanger sequencing. The pathogenic effect of the identified variants was confirmed using in silico prediction tools such as PolyPhen, MutationTaster, Varsome, etc. Additionally, the frequency of these variants in the general population was checked in ExAC, gnomAD, 1000 Genome Project databases, and + 2000 exomes/genomes. Additionally, segregation analysis of all the identified variants was performed using Sanger sequencing.

The phenotype in the patients presented here was mild to severe compared to previously reported cases from Saudi Arabia [31]. Indeed, the 16 patients previously reported presented with nephrolithiasis (13/16) and nephrocalcinosis (12/16). Among them, 4 patients revealed CKD stage 5, 3 underwent kidney transplantation, 4 showed nephrocalcinosis and nephrolithiasis and 3 with hyperoxaluria were asymptomatic [31]. In addition, they also observed urinary tract infection, abdominal pain, hematuria, and none of the patients presented an infantile form of PHI [31]. Furthermore, disease-causing *AGXT* variants [c.33dup; (p.Lys12GlnfsX156), c.187G > C; p.(Gly63Arg)] previously reported, and variants in patients presented here do not cluster within one domain and are distributed in different domains, which might be one of the reasons for heterogeneity in clinical presentation. Although, these regions are crucial for the normal function of *AGXT* and might be intolerant to different missense and frameshift variations reported in these regions. Consanguineous marriages are very common in Saudi Arabia, accounting for more than 52% of all marriages. Similarly, in the present study consanguineous marriages were observed in 88% of the patients. Interestingly, both consanguineous and non-consanguineous families revealed homozygous variants, showing that the variants are prevalent and hotspots in the Saudi population. The age of the affected individuals at first symptoms was between 1 month and 20 years, and the age at diagnosis was between 6 months and 39 years. In the cohort reported here, the average delay in diagnosis was approximately 3 years and 2 months.

Affected individuals diagnosed with any PHs should be treated immediately with high fluid intake. Urinary CaOx supersaturation can be reduced with alkali citrate treatment [32]. In the liver, citrate is converted to bicarbonate, and the alkali reduces the citrate reabsorption (intratubular) and thus citrate excretion is increased. For PH type I patients, the second treatment strategy includes enhancing the reduced activity of AGT [27]. As a small amount of urinary oxalate is derived from the diet in these patients, dietary oxalate restriction showed limited benefit [33]. In addition, individuals with PH1 have an excellent safety profile with pyridoxine (vitamin B_6_) treatment.

It was recently reported that the RNA interference (RNAi) therapeutic agent lumasiran targets the glycolate oxidase and thus reduces hepatic oxalate production. It has been reported to reduce urinary oxalate excretion, which is the main cause of kidney failure in PH1 patients. Most patients who received lumasiran treatment revealed normal levels within 6 months [34].

Such studies also highlight the need for periodic reevaluation of variants, either by molecular reanalysis, a certified laboratory, a geneticist, or scientists responsible for variant interpretation. The ACMG has issued standard guidelines for variant classification. A reasonable approach might be to review the molecular findings every 6 to 10 months if there is any reclassification of inherited VUS or likely pathogenic variant. This might help parents planning to have more children through pre-implantation genetic diagnosis (PGD) [35]. Additionally, population-specific allele identification will assist in data sharing among labs and ultimately decrease the burden. Molecular testing results are dynamic and significantly change with new variant reclassification over time that might help reduce VUS burden; however, a significant number of patients with inconclusive test results remain.

Physicians in KSA face many issues associated with disease management and treatment of rare genetic disorders. As the frequency of rare genetic disorders in KSA is more than in any other country, there is a need for a nationwide campaign that might raise awareness. KSA requires its own evidence-based nationwide guidelines for treating such rare disorders and physicians should be equipped with the latest knowledge about the latest therapies and new treatments approved by the FDA. However, prenatal genetic screening including noninvasive prenatal testing (NIPT), preimplantation genetic testing for aneuploidy (PGT-A), and preimplantation genetic testing for monogenic disorders (PGT-M) has been established recently in KSA to tackle such severe rare diseases [35, 36]. However, nationwide systematic neonatal screening and proper awareness is the need of the day [14].

Although we believe this study is the largest cohort from the region, it is limited by the relatively small sample size and the absence of functional studies. Nevertheless, we present clinical, molecular, and genetic data for 25 patients along with identification of common disease-causing variants found in the Saudi population. Such studies might help better understand the disease pathogenesis, which may facilitate evidence-based and well-designed therapies in the future.

## Supplementary Information

Below is the link to the electronic supplementary material.

Graphical Abstract (PPTX 45 KB)

## Data Availability

Upon reasonable request to the corresponding author.

## References

[1] Hoppe B (2012). An update on primary hyperoxaluria. Nat Rev Nephrol.

[2] Martin-Higueras C, Torres A, Salido E (2017). Molecular therapy of primary hyperoxaluria. J Inherit Metab Dis.

[3] Dindo M, Conter C, Oppici E, Ceccarelli V, Marinucci L, Cellini B (2019). Molecular basis of primary hyperoxaluria: clues to innovative treatments. Urolithiasis.

[4] Salido E, Pey AL, Rodriguez R, Lorenzo V (2012). Primary hyperoxalurias: disorders of glyoxylate detoxification. Biochim Biophys Acta.

[5] Williams EL, Acquaviva C, Amoroso A, Chevalier F, Coulter-Mackie M, Monico CG, Giachino D, Owen T, Robbiano A, Salido E, Waterham H, Rumsby G (2009). Primary hyperoxaluria type 1: update and additional mutation analysis of the AGXT gene. Hum Mutat.

[6] Cregeen DP, Williams EL, Hulton S, Rumsby G (2003). Molecular analysis of the glyoxylate reductase (GRHPR) gene and description of mutations underlying primary hyperoxaluria type 2. Hum Mutat.

[7] Bouzidi H, Majdoub A, Daudon M, Najjar MF (2016). Primary hyperoxaluria: a review. Nephrol Ther.

[8] Alfadhel M, Alhasan KA, Alotaibi M, Khalid Al Fakeeh KA (2012). Extreme intrafamilial variability of Saudi brothers with primary hyperoxaluria type 1. Ther Clin Risk Manag.

[9] Danpure CJ (2004). Molecular aetiology of primary hyperoxaluria type 1. Nephron Exp Nephrol.

[10] Cochat P, Liutkus A, Fargue S, Basmaison O, Ranchin B, Rolland MO (2006). Primary hyperoxaluria type 1: still challenging!. Pediatr Nephrol.

[11] Hoppe B, Dittlich K, Fehrenbach H, Plum G, Beck BB (2011). Reduction of plasma oxalate levels by oral application of Oxalobacter formigenes in 2 patients with infantile oxalosis. Am J Kidney Dis.

[12] Garrelfs SF, Rumsby G, Peters-Sengers H (2019). Patients with primary hyperoxaluria type 2 have significant morbidity and require careful follow-up. Kidney Int.

[13] Martin-Higueras C, Garrelfs SF, Groothoff JW, Jacob DE, Moochhala SH, Bacchetta J, Acquaviva C, Zaniew M, Sikora P, Beck BB, Hoppe B (2021). A report from the European Hyperoxaluria Consortium (OxalEurope) Registry on a large cohort of patients with primary hyperoxaluria type 3. Kidney Int.

[14] Alfadhel M, Umair M, Almuzzaini B, Alsaif S, AlMohaimeed SA, Almashary MA, Alharbi W, Alayyar L, Alasiri A, Ballow M, AlAbdulrahman A, Alaujan M, Nashabat M, Al-Odaib A, Altwaijri W, Al-Rumayyan A, Alrifai MT, Alfares A, AlBalwi M, Tabarki B (2019). Targeted SLC19A3 gene sequencing of 3000 Saudi newborn: a pilot study toward newborn screening. Ann Clin Transl Neurol.

[15] Alfadhel M, Almuqbil M, Al Mutairi F, Umair M, Almannai M, Alghamdi M, Althiyab H, Albarakati R, Bashiri FA, Alshuaibi W, Ba-Armah D, Saleh MA, Al-Asmari A, Faqeih E, Altuwaijri W, Al-Rumayyan A, Balwi MA, Ababneh F, Alswaid AF, Eyaid WM, Almontashiri NAM, Alhashem A, Hundallah K, Bertoli-Avella A, Bauer P, Beetz C, Alrifai MT, Alfares A, Tabarki B (2021). The leukodystrophy spectrum in Saudi Arabia: epidemiological, clinical, radiological, and genetic data. Front Pediatr.

[16] Alfadhel M, Abadel B, Almaghathawi H, Umair M, Rahbeeni Z, Faqeih EA, Almannai M, Alasmari A, Saleh M, Eyaid W, Alfares A, Mutairi FA (2022). HMG-CoA lyase deficiency: a retrospective study of 62 Saudi patients. Front Genet.

[17] Richards S, Aziz N, Bale S, Bick D, Das S, Gastier-Foster J, Grody WW (2015). Standards and guidelines for the interpretation of sequence variants: a joint consensus recommendation of the American College of Medical Genetics and Genomics and the Association for Molecular Pathology. Genet Med.

[18] Umair M, Khan MF, Aldrees M, Nashabat M, Alhamoudi KM, Bilal M, Alyafee Y, Al Tuwaijri A, Aldarwish M, Al-Rumayyan A, Alkhalaf H, Wadaan MAM, Alfadhel M (2021). Mutated VWA8 is associated with developmental delay, microcephaly, scoliosis and play a novel role in early development and skeletal morphogenesis in Zebrafish. Front Cell Dev Biol.

[19] Cochat P, Groothoff J (2013). Primary hyperoxaluria type 1: practical and ethical issues. Pediatr Nephrol.

[20] Cochat P, Hulton SA, Acquaviva C, Danpure CJ, Daudon M, De Marchi M, Fargue S, Groothoff J, Harambat J, Hoppe B, Jamieson NV, Kemper MJ, Mandrile G, Marangella M, Picca S, Rumsby G, Salido E, Straub M, van Woerden CS (2012). Primary hyerpoxaluria type 1: indications for screening and guidance for diagnosis and treatment. Nephrol Dial Transplant.

[21] Fargue S, Knight J, Holmes RP, Rumsby G, Danpure CJ (2016). Effects of alanine:glyoxylate aminotransferase variants and pyridoxine sensitivity on oxalate metabolism in a cell-based cytotoxicity assay. Biochim Biophys Acta.

[22] Monico CG, Olson JB, Milliner DS (2005). Implications of genotype and enzyme phenotype in pyridoxine response of patients with type I primary hyperoxaluria. Am J Nephrol.

[23] Hoyer-Kuhn H, Kohbrok S, Volland R, Franklin J, Beck BB, Hoppe B (2014). Vitamin B6 in primary hyperoxaluria 1: first prospective trial after 40 years of practice. Clin J Am Soc Nephrol.

[24] Bobrowski AE, Langman CB (2008). The primary hyperoxalurias. Semin Nephrol.

[25] Hoppe B, Beck B, Milliner DS (2009). The primary hyperoxalurias. Kidney Int.

[26] Harambat J, van Stralen KJ, Espinosa L, Groothoff JQ, Hulton SA, Cerkausiene R, Schaefer F, Verrina E, Jager KJ, Cochat P (2012). Characteristics and outcomes of children with primary oxalosis requiring renal replacement therapy. Clin J Am Soc Nephrol.

[27] Leumann E, Hoppe B (2001). The primary hyperoxalurias. J Am Soc Nephrol.

[28] Hopp K, Cogal AG, Bergstralh EJ, Seide BM, Olson JB, Meek AM, Lieske JC, Milliner DS, Harris PC, Rare Kidney Stone Consortium (2015). Phenotype-genotype correlations and estimated carrier frequencies of primary hyperoxaluria. J Am Soc Nephrol.

[29] Monico CG, Persson M, Ford GC, Rumsby G, Milliner DS (2002). Potential mechanisms of marked hyperoxaluria not due to primary hyperoxaluria I or II. Kidney Int.

[30] Belostotsky R, Seboun E, Idelson GH, Milliner DS, Becker-Cohen R, Rinat C, Monico CG, Feinstein S, Ben-Shalom E, Magen D, Weissman I, Charon C, Frishberg Y (2010). Mutations in DHDPSL are responsible for primary hyperoxaluria type III. Am J Hum Genet.

[31] Sanjad SA, Al-Abbad A, Al-Sabban E (1999). Primary hyperoxaluria type 1: an underestimated cause of nephrocalcinosis and chronic renal failure in Saudi Arabian children. Ann Saudi Med.

[32] Leumann E, Hoppe B, Neuhaus T (1993). Management of primary hyperoxaluria: efficacy of oral citrate administration. Pediatr Nephrol.

[33] Sikora P, von Unruh GE, Beck B, Feldkötter M, Zajaczkowska M, Hesse A, Hoppe B (2008). [13C2]oxalate absorption in children with idiopathic calcium oxalate urolithiasis or primary hyperoxaluria. Kidney Int.

[34] Garrelfs SF, Frishberg Y, Hulton SA, Koren MJ, O'Riordan WD, Cochat P (2021). Lumasiran, an RNAi therapeutic for primary hyperoxaluria type 1. N Engl J Med.

[35] Alyafee Y, Alam Q, Tuwaijri AA, Umair M, Haddad S, Alharbi M, Alrabiah H, Al-Ghuraibi M, Al-Showaier S, Alfadhel M (2021). Next-generation sequencing-based Pre-Implantation Genetic Testing for Aneuploidy (PGT-A): first report from Saudi Arabia. Genes (Basel).

[36] Alyafee Y, Al Tuwaijri A, Alam Q, Umair M, Haddad S, Alharbi M, Ballow M, Al Drees M, AlAbdulrahman A, Al Khaldi A, Alfadhel M (2021). Next generation sequencing based Non-invasive Prenatal Testing (NIPT): first report from Saudi Arabia. Front Genet.

