# Supplementary material for: Clinical and molecular characterization of a large primary hyperoxaluria cohort from Saudi Arabia: a retrospective study
Source: Pediatr Nephrol. 2022 Nov 21;38(6):1801–10. doi: 10.1007/s00467-022-05784-y (PMC10154271; doi:10.1007/s00467-022-05784-y)
Supplement: Supplementary file 1 — Graphical Abstract (PPTX 45 KB) [file 467_2022_5784_MOESM1_ESM.pptx]

## Slide 1
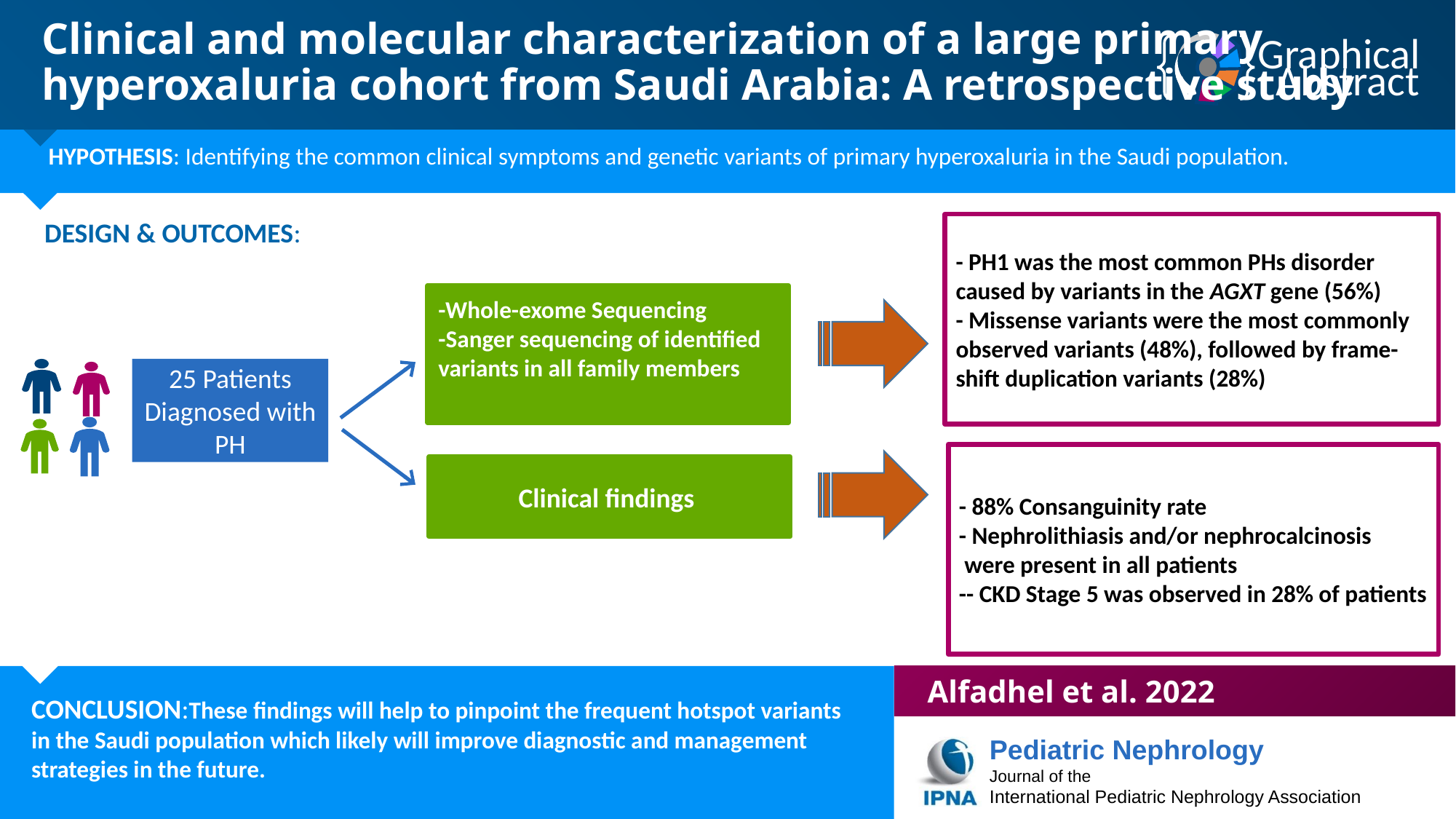

Clinical and molecular characterization of a large primary
hyperoxaluria cohort from Saudi Arabia: A retrospective study
HYPOTHESIS: Identifying the common clinical symptoms and genetic variants of primary hyperoxaluria in the Saudi population.
DESIGN & OUTCOMES:
- PH1 was the most common PHs disorder caused by variants in the AGXT gene (56%)
- Missense variants were the most commonly observed variants (48%), followed by frame-shift duplication variants (28%)
-Whole-exome Sequencing
-Sanger sequencing of identified variants in all family members
25 Patients Diagnosed with PH
- 88% Consanguinity rate
- Nephrolithiasis and/or nephrocalcinosis were present in all patients
-- CKD Stage 5 was observed in 28% of patients
Clinical findings
Alfadhel et al. 2022
CONCLUSION:These findings will help to pinpoint the frequent hotspot variants in the Saudi population which likely will improve diagnostic and management strategies in the future.
